# CRISPR-Cas13 in malaria parasite: Diagnosis and prospective gene function identification

**DOI:** 10.3389/fmicb.2023.1076947

**Published:** 2023-01-25

**Authors:** Elvis Quansah, Yihuan Chen, Shijie Yang, Junyan Wang, Danhong Sun, Yangxi Zhao, Ming Chen, Li Yu, Chao Zhang

**Affiliations:** ^1^Anhui Provincial Laboratory of Microbiology and Parasitology, Anhui Key Laboratory of Zoonoses, Department of Microbiology and Parasitology, School of Basic Medical Sciences, Anhui Medical University, Hefei, China; ^2^The Second Clinical Medical College, Anhui Medical University, Hefei, China; ^3^The First Clinical Medical College, Anhui Medical University, Hefei, China

**Keywords:** malaria, *Plasmodium*, CRISPR-Cas system, CRISPR-Cas13 diagnosis, CRISPR-Cas13 RNA editing

## Abstract

Malaria caused by *Plasmodium* is still a serious public health problem. Genomic editing is essential to understand parasite biology, elucidate mechanical pathways, uncover gene functions, identify novel therapeutic targets, and develop clinical diagnostic tools. Recent advances have seen the development of genomic diagnostic technologies and the emergence of genetic manipulation toolbox comprising a host of several systems for editing the genome of *Plasmodium* at the DNA, RNA, and protein level. Genomic manipulation at the RNA level is critical as it allows for the functional characterization of several transcripts. Of notice, some developed artificial RNA genome editing tools hinge on the endogenous RNA interference system of *Plasmodium*. However, *Plasmodium* lacks a robust RNAi machinery, hampering the progress of these editing tools. CRISPR-Cas13, which belongs to the VI type of the CRISPR system, can specifically bind and cut RNA under the guidance of crRNA, with no or minimal permanent genetic scar on genes. This review summarizes CRISPR-Cas13 system from its discovery, classification, principle of action, and diagnostic platforms. Further, it discusses the application prospects of Cas13-based systems in *Plasmodium* and highlights its advantages and drawbacks.

## 1. Introduction

Malaria has been a major global health problem for humans throughout their history and is a leading cause of mortality across many tropical and subtropical countries (Hemingway and Bates, 2003; Cowman et al., 2016; Monroe et al., 2022). Malaria control efforts have been undermined by the decline in the effectiveness of the primary malaria-figurehting tools, increasing resistance to treated-insecticide nets and anti-malarial drugs, and no effective vaccine (Olotu et al., 2013; Haldar et al., 2018; Memvanga and Nkanga, 2021). Spotlights from the 2022 world malaria report by WHO showed that cases of malaria increased from 245 million in 2020 to 247 million in 2021, although malaria-related deaths slightly decreased from 625,000 to 619,000 (World Health Organization, 2022). The etiological agent of malaria, *Plasmodium,* mainly thrives in human and mosquito hosts. Of all *Plasmodium* parasites, five species are responsible for global malaria infections in humans: *Plasmodium falciparum*, *Plasmodium vivax*, *Plasmodium malariae*, *Plasmodium ovale*, and *Plasmodium knowlesi* (Lalremruata et al., 2017; Memvanga and Nkanga, 2021; Sato, 2021).

Microscopic examination of Giemsa-stained peripheral blood smears has over the years been the “gold standard” for the diagnosis of malaria (Oboh et al., 2021; Fitri et al., 2022). However, its usefulness has been confronted with a host of challenges as results generated from microscopy are dependent on the technical know-how of the microscopist, the quality of blood sample, the parasite density, and the subjectivity of results interpretation (Barber et al., 2013; Berzosa et al., 2018; Ospina-Villa et al., 2018), indicating a void in diagnosis and the need to develop new effective tools. The *Plasmodium falciparum* genome encompasses 22.8 mega-bases with an average gene density of 1 gene per 4,338 base pairs—distributed among a total of 14 chromosomes (Gardner et al., 2002). Almost two-thirds of the proteins coded by these genes are unique to *Plasmodium* (Gardner et al., 2002) providing peculiar targets for molecular detection. In an attempt to fill the gaps in malaria diagnosis, several molecular diagnostic tools have been established to target various unique genes in *Plasmodium*. Molecular diagnosis notwithstanding remains a big huddle as the performance of these tools varies depending on the epidemiological setting (Oboh et al., 2018).

Interestingly, 74% of the total genes in *Plasmodium* remain functionally uncharacterized despite the availability of the parasite’s genome (Tan and Mutwil, 2020). To address this paucity, forward and reverse functional genomic tools have been developed to manipulate the parasites’ genomes to functionally characterize these genes (Zhang et al., 2014; Walker and Lindner, 2019; Cárdenas et al., 2022; Thiam et al., 2022). In particular, RNA-targeting tools hold great potential to understand the biological functions of genes and identify novel anti-malarial targets and effective vaccines (Nisbet et al., 2016; Liu et al., 2019). RNA level manipulation/editing affects the timing and/or level of transcription and alters the stability of gene expression, thereby offering scientists the platform to study the basic cellular functions of mRNAs, and non-coding RNAs (ncRNA) such as small nuclear RNA (snRNA), ribosomal RNA (rRNA), microRNA (miRNA), and piwi interaction RNA (piRNA; Briquet et al., 2022).

CRISPR (Clustered Regularly Interspaced Short Palindromic Repeats)-Cas (CRISPR-associated protein) system is found in archaea and bacteria—responsible for coordinating defense against foreign genetic elements (Mojica et al., 2000; Marraffini, 2015; Moon et al., 2019). The CRISPR-Cas system is a powerful biological tool whose usefulness scientists have been unlocking in the last few decades. It is a double-edged tool that scientists have applied for both diagnosis and gene editing to unravel the function of many genes at the DNA and RNA levels in several organisms (Graham and Root, 2015; Hartenian and Doench, 2015; Zhang D. et al., 2021). Currently, the CRISPR-Cas system is structurally and functionally diverse and divided into 2 classes, 6 types, and more than 30 subtypes. The first class includes type I, type III, and type IV, and the second class includes type II, type V, and type VI (O'Connell, 2019). The small size, atypical hypercompact architecture, and high efficiency of class 2 multi-domain Cas proteins have made them a prime focus of many researchers compared to class I proteins (Cao et al., 2022; Liu and Pei, 2022). Of these, the majority of research in the past decade has focused on CRISPR-Cas9 belonging to the type II category (Atkins et al., 2021), and its properties have been harnessed for gene knockdown, gene editing, epitranscriptomics modification, and sensing of RNA targets (Zhang H. et al., 2021). CRISPR-Cas13 systems—which constitute the type VI group—have been demonstrated to generally lack DNase (Deoxyribonuclease) activity, but are fixated on RNA cleaving, and could offer distinct targeting advantages to RNA. Thus they represent a clinically promising platform capable of efficiently characterizing genes at the RNA level (Gaj, 2021).

In this review, we have summarized the CRISPR-Cas13 system from its discovery, classification, principle of action, and its application in malaria diagnosis. Given that malaria parasites lack relevant interference systems (Baum et al., 2009) thereby limiting the use of endogenous interference-dependent RNA editing systems, we will further discuss the application prospects of Cas13 in *Plasmodium* at the RNA level.

## 2. Discovery of CRISPR-Cas systems, especially for Casl3

In 1987, Yoshizumi Ishino of Osaka University in Japan detected regularly spaced direct palindromic repeats downstream of the stop codon of the iap (isoenzymes of alkaline phosphate) genes of *E. coli* K-12 cells (Ishino et al., 1987). This event provided the blueprint for the discovery of new CRISPR-Cas systems, ushering scientists into a new era of gene editing and genomic engineering. At the time, the relevance of these repeat sequences remained obscure. Mojica and his team detected a similar sequence in phylogenetically distinct organisms: *Haloferax mediterranei* and *Haloferax volcanii* of the Archaea domain (Mojica et al., 1993). They later postulated that these repeats are involved in replicon partitioning (Mojica et al., 1995). In 2002, substantial progress was made by Jansen and collaborators resulting in the official naming of this mysterious repeat as CRISPR which is now commonly accepted by the scientific community (Jansen et al., 2002). Soon after, spurred on by his previous findings, a major leap toward the understanding of the function of CRISPR was made by Mojica and his team (Mojica et al., 2005). They pointed out that the spacer sequence in CRISPR had homology with foreign phages or plasmids, and that the virus could not infect cells carrying homologous spacer sequences, but was easy to invade cells without spacer sequences. Against this background, it was posited that CRISPR might participate in the immune function of bacteria and it requires a precise sequence match between the spacer and the target sequence of the virus (Mojica et al., 2005; Lander, 2016). The functionality of the second component of the CRISPR-Cas complex, CRISPR-associated protein (Cas), was computationally demonstrated later by Marakova and collaborators as a composite of the DNA repair machinery (Makarova et al., 2006). In a later study by Horvath, he noticed a palindromic repeat that coded for an endonuclease protein, Cas9, and intimated that the protein cleaves viral DNA and thus grants immunity to the host bacteria (Horvath and Barrangou, 2010). A few years later, in 2012, Doudna and Charpentier’s team discovered the third component of the CRISPR system—the trans-acting crRNA (tracrRNA) which oversees the maturation of precursor crRNA into crRNA and also directs Cas9 cleavage of dsDNA (Jinek et al., 2012). Subsequent development saw the exploitation of the CRISPR-Cas system as a genomic editing tool. In 2013, the gene-editing tool CRISPR-Cas9 was applied in mammalian cells and became popular all over the world, because of its novelty, efficiency, and versatility (Shmakov et al., 2015).

**Figure 1:**
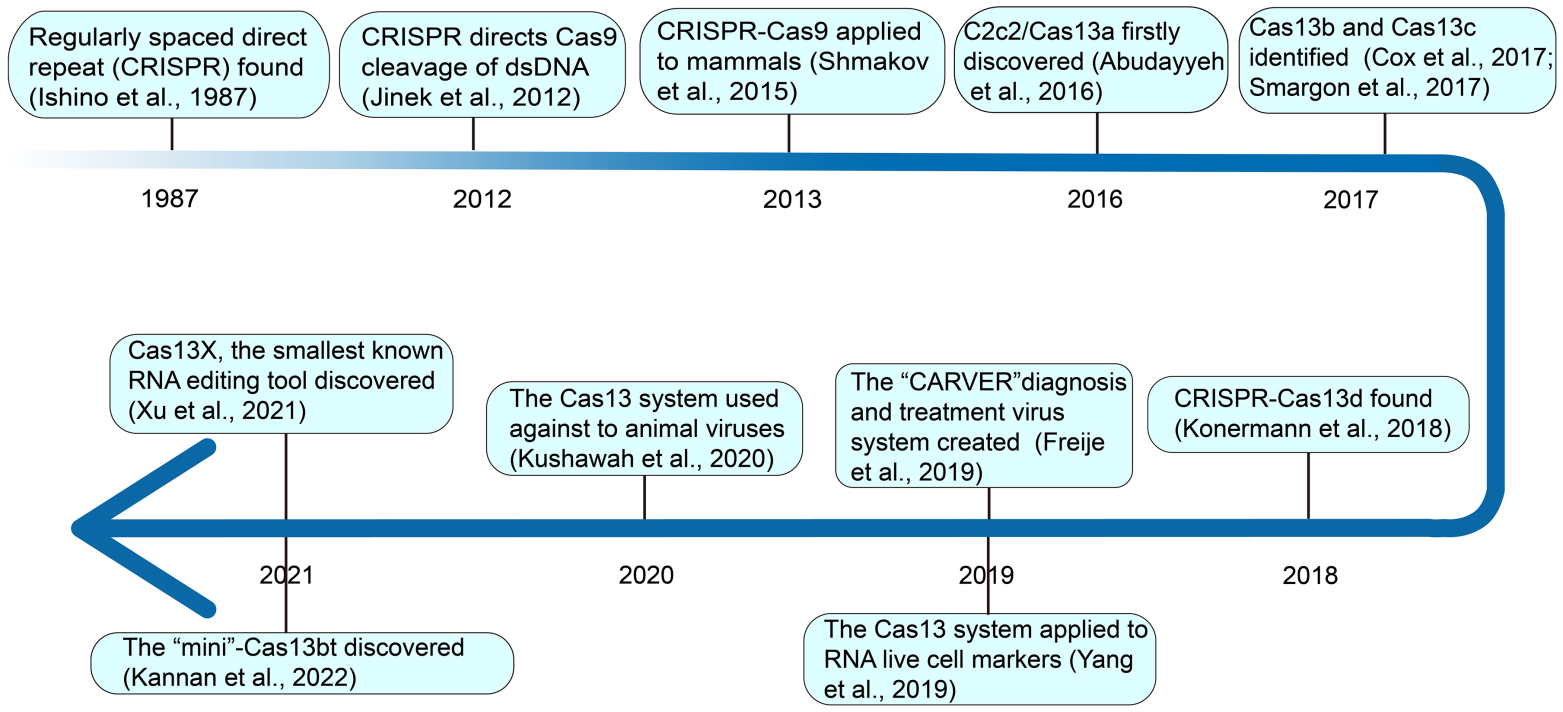
The discovery and application timeline of CRISPR-Cas13 system.

Different from CRISPR-Cas9, which binds and cuts at the DNA level, as shown in Figure 1, in 2016, deeply searching for microbial genome data, it was found that a new CRISPR-Cas system effector protein, named c2c2 (now referred to as Casl3a), could specifically bind and cut RNA under the guidance of crRNA, and regulate the activity of genes at the RNA level (Abudayyeh et al., 2016). This opened up a new avenue for editing targeted RNA. In 2017, through rigorous biochemical experiments and genetic computations, Zhang Feng’s team identified two new Cas13 subtypes, Cas13b and Cas13c (Cox et al., 2017; Smargon et al., 2017). Subsequent investigation revealed that CRISPR-Cas13 systems uniquely lack the third component, tracrRNA (Yan et al., 2019). Going further, they demonstrated that Cas13b could be applied to precisely edit the full length of transcript harboring deleterious mutations in mammalian cells (Cox et al., 2017). In 2018, Konermarm et al. analyzed a prokaryote genome and discovered the CRISPR-Casl3d system, which has the smallest molecular weight of all CRISPR-Casl3 effectors, and was suitable for virus vectors, and had great application potential *in vivo* (Konermann et al., 2018). Through the exploration of bulk metagenomic data, Hu et al. (2022) identified novel hypercompact Cas13 systems and therein named them Cas13e, Cas13f, Cas13g, Cas13h, and Cas13i. However, their functional relevance remains to be described in future studies.

Since its advent, Cas13 has been widely used in various fields. For example, in 2019, Zhang Feng’s team created a new CRISPR-Cas13 system called CARVER (Cas13-assisted Restriction of Viral Expression and Readout) antiviral, which combines the antiviral activity of Cas13 and its diagnostic capabilities, as a promising system for the diagnosis and treatment of viral infections (Freije et al., 2019). In the same year, Chen Lingling’s group used the CRISPR-Casl3d system for RNA live cell labeling (Yang et al., 2019). In 2020, Gopal Kushawah and others used CRISPR-Cas13d to induce high-efficiency nucleoprotein degradation in animal embryos, providing new conditions for animal antiviral research (Kushawah et al., 2020). More recently, in 2021, Yang Hui’s team identified Cas13X and Cas13Y from high-salinity samples and designed an RNA interference experiment for Cas13X.1 in a mammalian cell line (Xu et al., 2021). During the same period, Zhang Feng’s team discovered “Mini”-Cas13bt, functionalizing Cas13bt through the use of adenosine and cytosine deaminase (Kannan et al., 2022).

**Figure 2:**
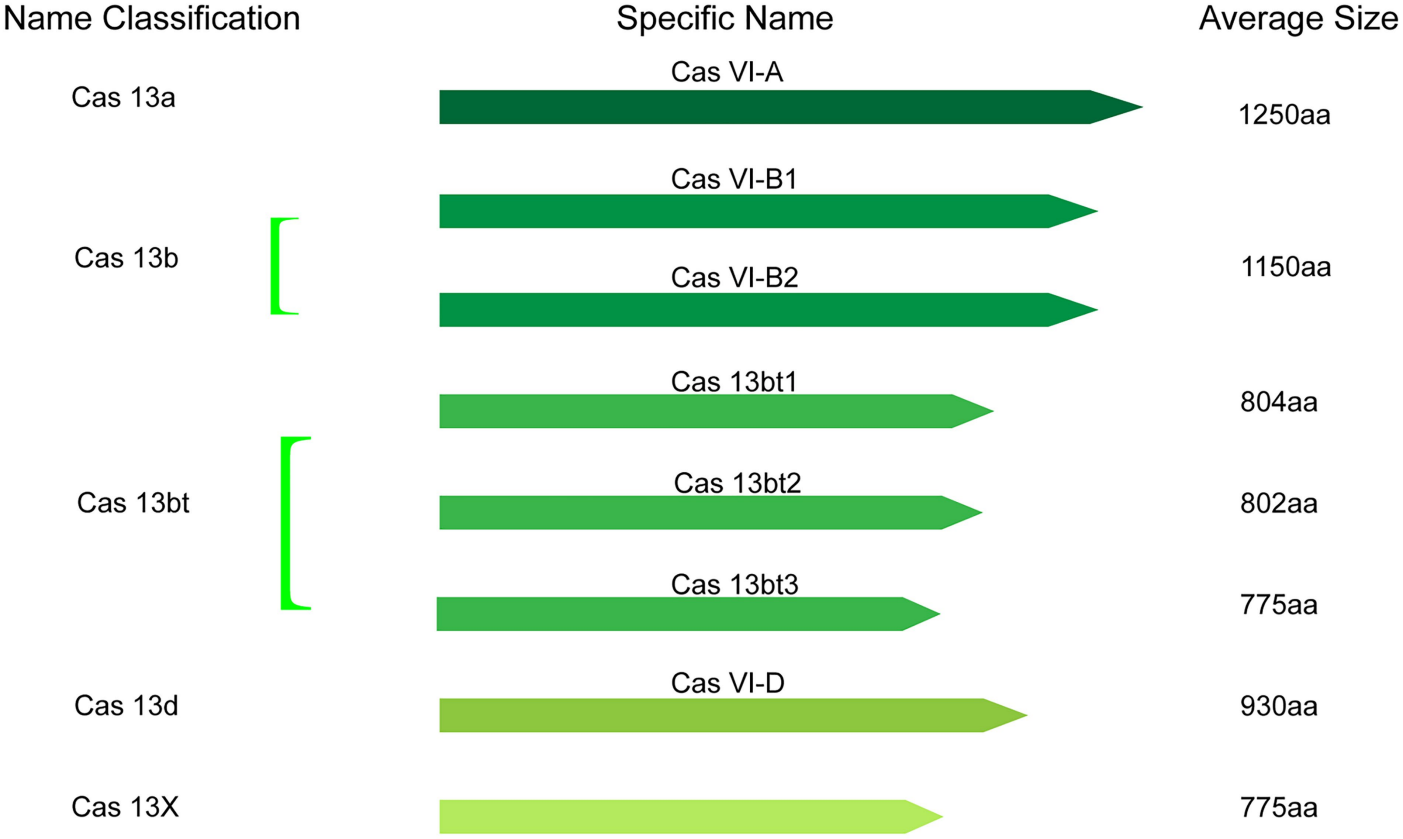
The classification and average size of Cas13 proteins with having been widely used in various fields.

Collectively, the catalog of CRISPR-Cas13 systems keeps expanding as novel types and orthologs are being described in current studies. At present, based on phylogeny, this important class of effector proteins is categorized into VI-A (Cas13a), VI-B (Cas13b), VI-C (Cas13c), VI-D (Cas13d; Zhang H. et al., 2021), VI-E to I (Cas13e-Cas13i; Hu et al., 2022), and the latest CRISPR-Cas13 system like Cas13X, Cas13Y (Xu et al., 2021) and Cas13bt (Kannan et al., 2022). Here, the classification and average size of Cas13 proteins, which have been widely used in various fields, were summarized (Figure 2).

## 3. The components of the CRISPR-Cas13 system

**Figure 3:**
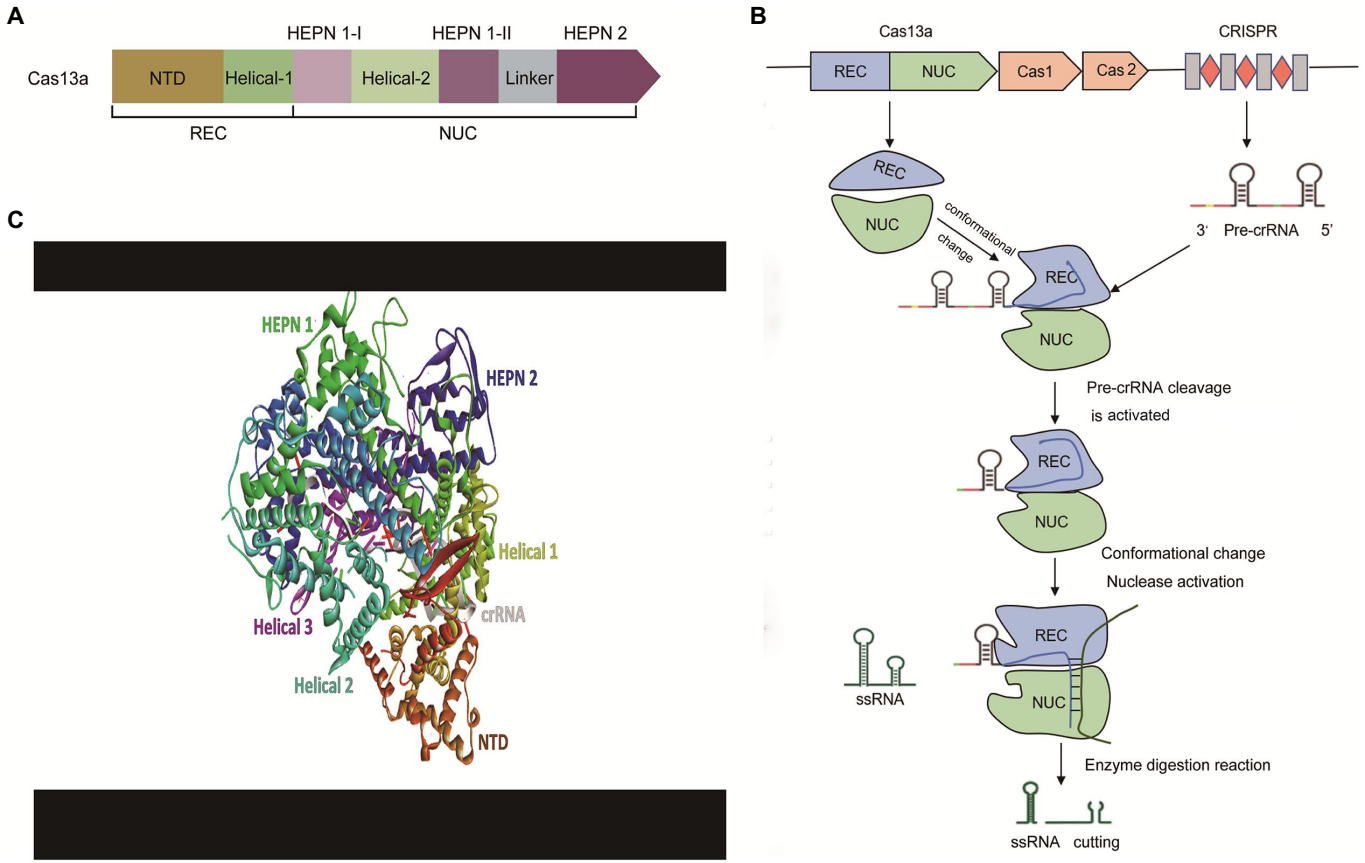
The effector modules and mechanism of action of the CRISPR-Cas13a. **(A)** The architecture of effector modules of Cas13a. Cas13a has a bi-lobed structure comprising a ‘Nuclease’ (NUC) lobe and a ‘Recognition’ (REC) lobe. NUC Lobe contains two distinct but functionally linked endonuclease sites—Higher Eukaryotes and Prokaryotes Nucleotide-binding Domain 1 and 2 (HEPN-1 and HEPN-2) separated by a basic linker/Helical-3 domain, and a Helical-2 domain. The REC lobe encompasses the N-terminal domain (NTD) and the Helical-1 domain, **(B)** The mechanism of action of the CRISPR-Cas13a. The pre-crRNA recognizes and binds to the REC leaves of Cas13a. Next, Cas13a processes its pre-crRNA to form a stable crRNA-Cas13a complex. Subsequently, the target ssRNA is recruited to the crRNA-Cas13a complex to undergo base-complementary pairing with the matured crRNA resulting in the activation of the Cas13a endonuclease enzyme. Finally, the activated catalytic domain of Cas13cleaves the target sequence, and **(C)** Crystal structure of Cas13a complex with crRNA. REC, Recognition lobe; NUC, Nuclease lobe; CRISPR, Clustered Regularly Interspaced Short Palindromic Repeats; Pre-crRNA, Precursor CRISPR RNA; ssRNA, Single-stranded RNA.

As shown in Figure 3, using Cas13a as an example, the CRISPR-Cas13 system mainly consists of crRNA (CRISPR RNA) and CRISPR-related nucleases (Cas1, Cas2, and Cas13). The biology of these components is briefly discussed below.

### 3.1. CRISPR-related nuclease (Cas13)

Cas13a has a bi-lobed structure comprising a ‘Nuclease’ (NUC) lobe and a ‘Recognition’ (REC) lobe. These two domains form a cavity that accommodates the crRNA. NUC Lobe contains two distinct but functionally linked endonuclease sites—Higher Eukaryotes and Prokaryotes Nucleotide-binding Domain 1 and 2 (HEPN-1 and HEPN-2) separated by a basic linker/Helical-3 domain, and a Helical-2 domain (Zhu and Huang, 2018; O'Connell, 2019; Figure 3A). The REC lobe encompasses the N-terminal domain (NTD) and the Helical-1 domain (Liu and Pei, 2022). The HEPN-1 acts as a scaffold that links NUC and REC lobes (Zhang et al., 2018). The formation of the spacer-protospacer binary complex is driving the recognition of the stem-loop (5’handle) of crRNA by NTD. In Cas13d effectors, the α1 component of the HEPN1 and the C-terminal of HEPN-2 domains form a catalytic site that primes the spacer-protospacer binary complex for both targeted and collateral cleavage (Zhang et al., 2018). The 5′ end repeat region of crRNA binds to the REC leaf so that the guide region sequence of crRNA is guided into the cavity, formed in the NUC area (nuclease lobe). The NUC leaf has two different domains, NUC1 and NUC2. The function of these two domains is to “sandwich” the guide region of crRNA, thereby forming a plane, allowing the complementary sequence of the target ssRNA (single-stranded RNA) to bind for cleavage.

### 3.2. CRISPR RNA

A typical crRNA landscape includes a promoter within an AT-rich leader sequence adjacent to the first CRISPR repeat (Jiang and Doudna, 2015). CRISPR RNA (crRNA) is synthesized from the transcriptions of the CRISPR array into a long precursor crRNA (pre-crRNA) and subsequently processed enzymatically by removing the repeat region and part of the spacer sequence into a smaller matured crRNA (Kenchappa et al., 2013; Jiang and Doudna, 2015). The crRNA is composed of a single variable spacer flanked by short direct CRISPR repeat sequences at the 5′ and 3′ ends. The 5′ end direct repeat (5′ handle) morphs into a short hairpin loop (handle) with a guide segment which when in complex with Cas13 proteins is sandwiched by the HEPN2 of the Nuclease domain and NTD of the Recognition domain (Zhu and Huang, 2018). Downstream, the hairpin loop is a 3–5 nucleotide sequence which promotes firm binding and Cas13 catalytic activities (O'Connell, 2019). The spacer region of crRNA is clamped between the Helical-1 and Helical-2 domains (Zhang et al., 2018). Embedded at the center of the 3′ end of the guide sequence is a “seed” region that directs the scanning-for-target process and ensures firm hybridization with the protospacer of target RNA (Zhu and Huang, 2018; Bandaru et al., 2020). Mismatch at the middle of the seed region of Cas13a maximally reduces the binding affinity, while mismatches elsewhere result in a subtle reduction in binding affinity (Tambe et al., 2018). Thus precise complementarity, particularly in the middle of the seed region, is key for target binding and subsequent cleavage. Of importance, a single base-pair mismatch is sufficient to attenuate Cas13a HEPN activation despite the firm binding affinity between crRNA and target RNA (Tambe et al., 2018).

## 4. The mechanism of action of the CRISPR-Cas13 system

During bacteria immune surveillance and cleavage of exogenous RNA, CRISPR-Cas13 requires a guide from a matured crRNA (Bayoumi and Munir, 2021; Krohannon et al., 2022). At a glance, this seems a challenge since CRISPR type V1 lacks Cas6 or Cas5d endonuclease activity for processing pre-crRNA into matured crRNA (crRNA biogenesis). Now, it has been shown that Cas13 performs both crRNA biogenesis and RNA cleavage employing two chemically and mechanistically distinct mechanisms for both tasks (East-Seletsky et al., 2016). Using three purified recombinant Cas13 proteins, it was revealed that Cas13 cut at two to five nucleotides upstream of the target pre-crRNA to produce a 60–66 nucleotide-long matured crRNA (Huynh et al., 2020). The resulting matured crRNA contains a single spacer sequence (20–30 nucleotide long) that is complementary to the target RNA sequence and a direct repeat region (Ai et al., 2022).

A generalized molecular action of CRISPR-Cas13 is briefly described in Figures 3B,C. CRISPR-Cas13-mediated cleavage is initiated as pre-crRNA recognizes and binds to the REC leaves of Cas13 to form an intermediate transition state complex. This event induces a conformational change in the conserved residues between Helical-1 and HEPN2 in the NUC region (Zhang et al., 2019). HEPN-2 provides an acid–base catalytic site that catalyzes the processing of pre-crRNA into a matured crRNA (Zhang et al., 2019). Upon complementary pairing between the matured crRNA and target ssRNA, a conformational rearrangement is triggered, as the catalytic HEPN domain of HEPN-2 moves close to the catalytic HEPN domain of HEPN-1 (Zhang et al., 2019). The two HEPN domains combine to form a single catalytically competent active site—which subsequently cleaves the RNA target sequence (Makarova et al., 2017; O'Connell, 2019).

### 4.1. The collateral damage pitfall of Cas13 system

The utility of CRISPR-Cas13 has been hampered by its promiscuous degradation of unintended RNA targets by the active HEPN domain resulting in collateral damage (Abudayyeh et al., 2016; Smargon et al., 2017; Yan et al., 2018; Ai et al., 2022). This setback seems not typical of all Cas13 systems, but it has been observed in Cas13a of *Leptotrichia shahii* (Shmakov et al., 2015) and *Leptotrichia buccalis* (East-Seletsky et al., 2016). It is expected that any efficient Cas13-based editing platform should invariably eliminate the Cas13 off-target activity to increase specificity. To avert this undesirable property, different engineering strategies have been adopted. In one recent study, the truncation of the crRNA spacer length led to the loss of off-target catalytic activities but maintained target binding specificity, and this property was harnessed to effectively knock down multiple transcripts with reduced collateral damage (Abudayyeh et al., 2017). In a different study, transgenic constructs of cas13 (a-d) variants with altered codons were shown to be specific with very low tolerance to off-RNA targets but high fidelity in Drosophila SG4_CD cells (Huynh et al., 2020). Interestingly, Lin et al. have discovered and described novel anti-CRISPR-Cas13a inhibitors (arcVI-A) capable of attenuating RNA targeting and editing in human cells. The application of these molecules could modulate precise RNA editing and also inhibit unintended cleavage (Lin et al., 2020). Altogether, it appears that the off-target property of Cas13 effector proteins is amendable and thus could be efficiently used to edit RNA targets.

## 5. CRISPR-Cas13 system as a diagnostic tool

**Figure 4:**
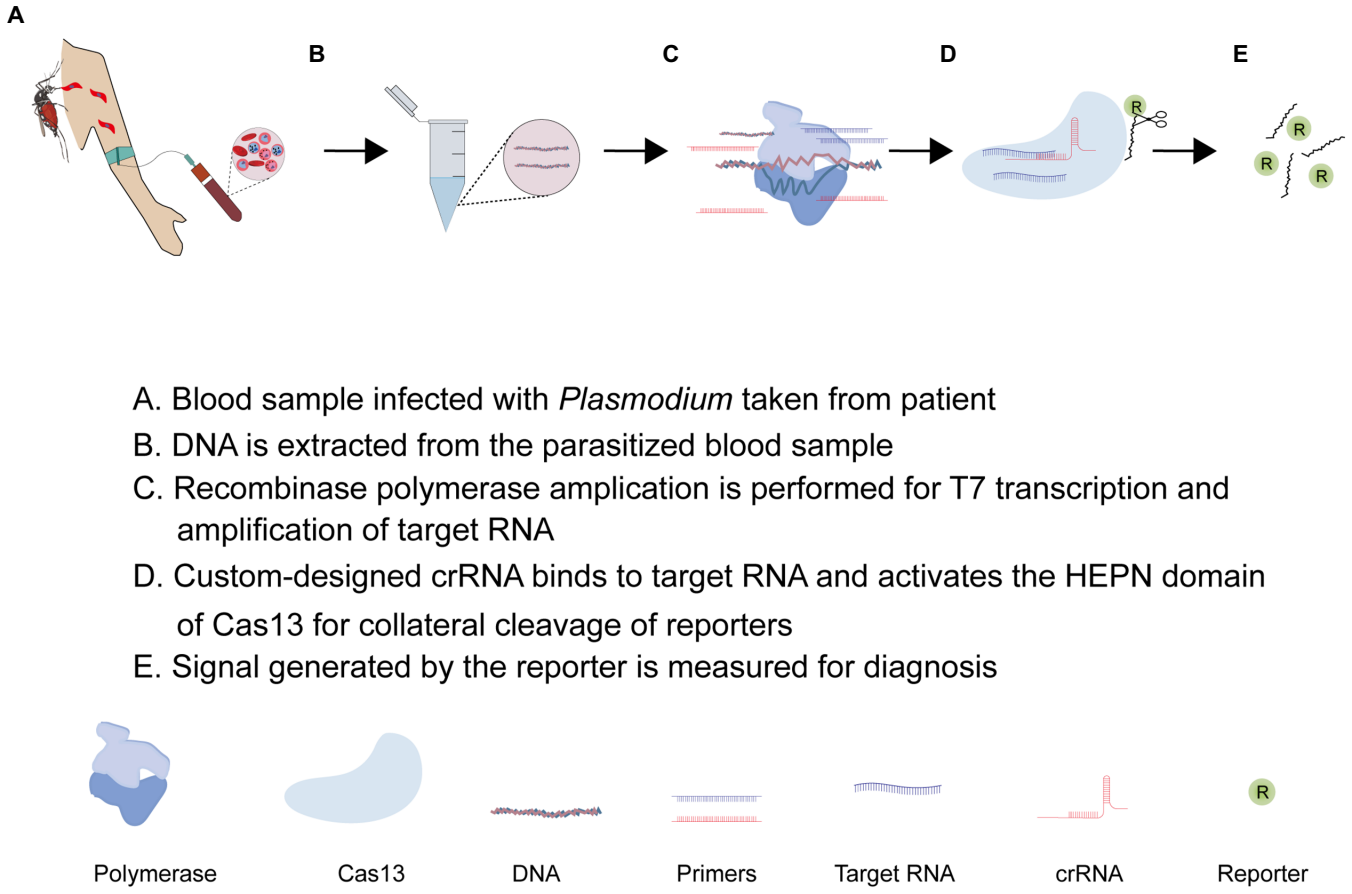
The flow chart of CRISPR-Cas13 genetic testing tool “SHERLOCK.”

As described earlier, the promiscuous nuclease activity of some Cas13 systems possess a central challenge in their application. This challenge is not essentially negative but opens up new ways for nucleic acid detection if it is carefully harnessed and appropriately applied. This is what the recently developed SHERLOCK platform does. As shown in Figure 4, the SHERLOCK CRISPR tool combines an isothermal nucleic acid amplification technique, and recombinant polymerase amplification technique (RPA), and leverages the indiscriminate endonuclease activity of Cas13 effector protein for clinical diagnosis of diseases (Kellner et al., 2019; Mahas et al., 2021; Mustafa and Makhawi, 2021). While many Cas13 orthologs have been identified and functionally characterized in different bacteria, LwCas13a is the most widely used type in the SHERLOCK applications (Patchsung et al., 2020). Recent advances have seen LwCas13a-based SHERLOCK adopted in different ways (Patchsung et al., 2020). Regardless of the approach, the Cas13-based SHERLOCK platform essentially makes use of: (1) target sequence found in the test sample; (2) T7 RNA polymerase promoter and T7 RNA polymerase for RPA; (3) RNA reporters or sensors sensitive to Cas13 collateral activities; and (4) custom-designed crRNA linked to a programmed Cas13 effector protein for guided detection of target RNA and collateral cleavage of reporters, respectively. The SHERLOCK protocol usually starts with the pre-amplification of the DNA/RNA using RPA primers and the conversion of target dsDNA into ssRNA using the T7 transcriptase enzyme. This is followed by the detection of RNA targets with custom Cas13-crRNA. The custom-designed crRNA is generally composed of a variable spacer region complementary to the target RNA, a constant region that recruits Cas13a, and a T7 promoter sequence that enhances *in vitro* transcription (Gootenberg et al., 2017; Kellner et al., 2019). Once the spacer of the crRNA aligns with the target RNA, the constant region binds to the Cas13 effector protein resulting in the activation of the off-target HEPN endonuclease activity of Cas13 which consequently cleaves an RNA-tagged reporter to generate a signal for detection (Rajan et al., 2022). To improve signal generation, Yang et al. (2022) engineered a LwCas13a with improved collateral activity by inserting different RNA-binding domains into a unique site within the HEPN domain. The SHERLOCK application has been developed into a cheap and convenient paper-based CRISPR-Cas13-base diagnosing assay capable of detecting and differentiating RNAs of viruses (Zika virus and Dengue virus) and genotyping DNA of bacteria (*Escherichia coli* and *Pseudomonas aeruginosa*; Gootenberg et al., 2017; Myhrvold et al., 2018; Manning et al., 2021).

### 5.1. CRISPR-Cas13-based diagnosis of *Plasmodium* infection

Besides Cas13a, Class II type V CRISPR-Cas12a (cpf1) has been described to possess a weak collateral activity making it useful in detecting target ssDNA or dsDNA (Nguyen et al., 2020; Kim et al., 2021; Lv et al., 2021; Wei et al., 2021). A Cas12-based SHERLOCK platform has been employed in detecting and differentiating *Plasmodium* species. In 2020, Lee and his team adopted this application to detect and distinguish *P. falciparum* (Pfr364 gene)-, *P. vivax* (18s rRNA)-, *P. ovale* (18s rRNA)-, and *P. malaria* (18s rRNA)*-*specific dsDNA using programmed Cas12a (cpf1) effector proteins (Lee et al., 2020). Noteworthy, the T7 transcription step is skipped, allowing for the direct detection of amplified targeted DNA instead of RNA. This assay could detect less than two parasites per microliter of blood and showed high sensitivity and specificity in differentiating clinical samples of *P. falciparum* and *P. vivax* with 100% accuracy. Based on these data, a field-applied diagnostic method for asymptomatic carriers was established for a rapid clinical diagnosis of non-*falciparum* malaria and low-density *P*. *falciparum* infections (Lee et al., 2020). Of notice, their fluorescence read-out assay demonstrated a relatively low fluorescence signal at 50 attomolar (30 parasites per microliter) possibly due to the weak collateral activity of Cas12a. Against this backdrop, the strong collateral activity of Cas13a provides an appropriate alternative to efficiently detect *Plasmodium* infections using the Cas13-based SHERLOCK platform. An additional advantage of Cas13 is its auto-catalytic ability to process its pre-cRNA without the involvement of tracrRNA and thus allows for the use of multiple guide crRNA in a single streamlined multiplex assay. Accordingly, these properties have been leveraged for a triple-purpose assay involving *Plasmodium* detection, species differentiation, and drug resistance genotyping using custom-designed crRNA with spacers specific to *P. falciparum*, *P. vivax,* and *dhps* (dihydropteroate synthetase) variants in a single multiplex assay (Cunningham et al., 2021). In briefly, 30–35 nucleotide-long RPA primers tagged with T7 promoter sequences were used to amplify *a dhps* sequence and/or *Plasmodium* 18S ribosomal RNA sequence conserved across human-infecting *Plasmodium* species to generate a short dsDNA. The assay utilized a custom-designed 67 nucleotide-long crRNA composed of a variable spacer region complementary to the target RNA, a constant region that recruits LwCas13a, and a T7 promoter sequence to enhance *in vitro* transcription. Using T7 polymerase, the resulting short dsDNA of the target sequence is transcribed into ssRNAs *in vitro*. Once the spacer of the crRNA aligns with the target RNA, the constant region binds to LwCas13a resulting in the activation of the off-target endonuclease activity of LwCas13a which consequently cleaves a fluorescent or colorimetric reporter RNA to generate a signal. Predefined nucleotide variations in *dhps* are known to confer resistance to sulfadoxine or sulfadoxine-pyrimethamine (SP; Okell et al., 2017), which are the main anti-malarial drug for intermittent preventive treatment in pregnant women. Thus, when the Cas13a-based SHERLOCK platform is improved to a point-of-care diagnostic tool, it will inevitably benefit the quest to track the continuous evolution of anti-malarial drug resistance in endemic areas.

## 6. Gene manipulating tools at the RNA level in *Plasmodium*

Most eukaryotic organisms possess an evolutionarily conserved mechanism—RNAi, which regulates genes post-transcriptionally and guards against intrinsic and extrinsic threats by degrading the coding regions of target genes. Typical RNAi-related genes such as Dicer and Argonaute have been identified in *Plasmodium*, but they lack endogenous RNAi machinery, which hinders gene annotation (Mueller et al., 2014). In this regard, non-canonical RNAi systems have been designed to target RNAs in *Plasmodium*. In 2020, Franziska Hentzschel’s team designed the rodent *Plasmodium berghei* to express a minimal, unconventional RNAi mechanism that only requires Ago2 (argonaute 2) and a modified short loop RNA, AgoshRNA. The non-canonical AgoshRNA for target genes structurally encompasses a 5 bp hairpin loop (CTTCA) with a sense sequence (having G-U and G-C mismatches initial and terminal codons, respectively) and an antisense sequence attached to either end of the construct. By integrating an AgoshRNA episomally maintained in plasmid into an RNAi-competent *Plasmodium*, they achieved an efficient gradient gene-knockout of several genes although its application is currently limited to the erythrocytic-stage parasites (Hentzschel et al., 2020).

To circumvent the occurrence of cell death when essential genes are detected for editing/manipulation, conditional manipulating tools such as glms ribozyme system and the Tet operating system have been designed to manipulate the RNA of *Plasmodium* (Prommana et al., 2013; Briquet et al., 2022). In Tet systems, the promoter of the target gene is replaced with unstable multiple tetracycline operating sites that bind to a transactivator domain for transcription. This system can be turned off or on depending on the presence or absence of anhydrotetracycline (de Koning-Ward et al., 2015). The glms/riboswitch system is designed to integrate an auto-cleaving ribozyme gene into the target gene sequence which upon expression cleaves the UTR region of the target mRNA to induce degradation (Prommana et al., 2013; de Koning-Ward et al., 2015).

### 6.1. RNA editing advantages of Cas13

In the last decade, steady progress has been made in the use of CRISPR-Cas13 systems to systematically interrogate the genome of several different organisms. For instance, Cas13 (Cas13a, Cas13d, cas13X, and cas13Y) RNAi systems have been applied for functional characterization of various genes in zebrafish embryos, medaka embryos, mouse embryos, and other mammalian cells demonstrating their practicability in varied cell lines (Cox et al., 2017; Konermann et al., 2018; Kushawah et al., 2020; Xu et al., 2021). Although the CRISPR-Cas13-based system has not been widely applied to RNAs of *Plasmodium* it would be of great importance if adapted to *Plasmodium* owing to the advantages discussed below.

The CRISPR-Cas9 system is by far the most established CRISPR-Cas system for interrogating *Plasmodium*’s genome at the DNA level (Crawford et al., 2017; Nishi et al., 2021). Based on the observation that the HNH catalytic domain of Cas9 is homologous to RNA-cleaving HNH, Cas9 has been repurposed to cleave RNA targets (O’Connell et al., 2014) adding to its already known DNA cleaving property. It has however been shown that the binding affinity and programmable cleaving of target RNA by Cas9 require the presence of sequence-specific PAM-presenting oligonucleotide as a separate DNA, an additional component that is not needed when using Cas13 (O’Connell et al., 2014). In the absence of PAM-presenting oligonucleotides, Cas9 only exhibits steric inhibition of protein translation with no obvious effect on mRNA architecture or levels indicating a limitation to its transcript editing prospects (Liu et al., 2016). Like Cas13, Cas9 is also limited by its unexpected off-target effects (Fu et al., 2013, 2014; Yang et al., 2014) although there have been attempts to experimentally avert this undesirable catalytic property (Fu et al., 2014; Kleinstiver et al., 2016; Slaymaker et al., 2016). Due to the affinity of HNH and RuvC domains of Cas9 to DNA, off-target mutagenesis by Cas9 could imprint unwanted permanent genomic scars on genomic DNA sites having corresponding RNA PAMs or any proximal endogenous locus (Haeussler et al., 2016; Ayabe et al., 2019), which may hinder attempts to associate intended genetic edits to observed experimental results. Cas13 is not constrained by PAM sequence at the target locus (Cox et al., 2017; Tang et al., 2021), making it a flexible tool to use compared to Cas9. Another impediment to the use of Cas9 is that, for DNA targets, edits cannot be made distal (50–100 bp away from) to the point of cut (Elison and Acar, 2018). It is however unknown if this limitation also exists during RNA editing with Cas9. Another peculiar RNA editing advantage of Cas13 is its auto-catalytic ability of Cas13 to process its pre-crRNA, which allows for streamlined delivery of targeting crRNAs for large-scale mRNA transcript editing without loss of specificity and efficiency (Gootenberg et al., 2018; Ackerman et al., 2020; Li et al., 2022; Thakku et al., 2022). This property has already been applied to manipulate a host of genes in live cells (East-Seletsky et al., 2016).

Although the already discussed RNAi systems (Dicer, Argonaute, Tet, and glms ribozyme systems) have great potential, Cas13-based RNA systems have advantages: (1) it does not depend on the cellular RNAi machinery; (2) it is not prone to toxicity from exogenous additives (as in the case of the use of glucosamine-6-phosphate in the glms system); and (3) it can be used in strains the lacks inducible expression systems.

Cas13-based systems however have drawbacks. Firstly, many investigators have shown that the effectiveness of Cas13a, Cas13b, and Cas13d is dependent on the length of crRNA (Abudayyeh et al., 2017; Bandaru et al., 2020; Huynh et al., 2020). It has been demonstrated that the ideal crRNA length is 23–30 nucleotides and any guide shorter or longer may have compromised effectiveness (Abudayyeh et al., 2017; Bandaru et al., 2020; Huynh et al., 2020). Secondly, it has been revealed that crRNA designed to target intronic splice sites or 3’ UTR regions activates lower cleavage activity relative to crRNAs targeting coding regions (Wessels et al., 2020). Thus, an improvement on the combined HEPN catalytic activity of HEPN-1 and HEPN-2 may be required when targeting splice sites or 3’ UTR regions. Thirdly, effective cleavage of mRNAs of essential genes may be toxic to the host cell (Abudayyeh et al., 2016). Lastly, the delivery of less compact Cas13 effectors is derailed by the size of the RNA editors as they exceed the loading capacity of the adeno-associated virus vectors (Hu et al., 2022). Thus, the size of Cas13 effectors is a critical factor to consider in future applications of the CRISPR-Cas13 genomic tool in *Plasmodium*.

### 6.2. Prospects of Cas13 for manipulating RNAs in *Plasmodium*

**Figure 5:**
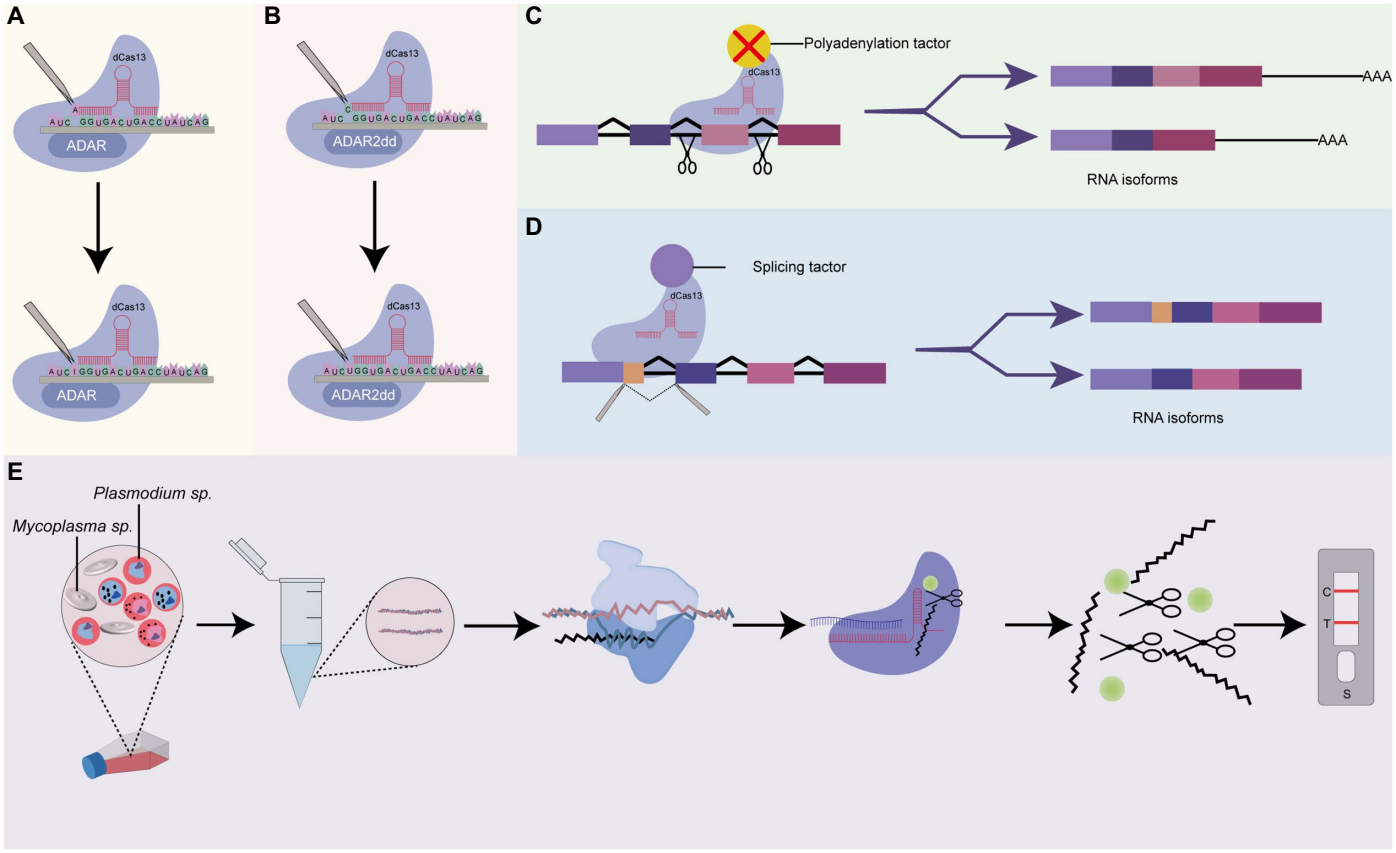
Prospects of Cas13-base systems for RNA editing in *Plasmodium*. **(A)** dCas13 tethered to ADARs for a single-nucleotide base editing in RNA. This system uses dCas13 effector-deaminase acting on RNAs (ADARs) domains fusion, for crRNA-guided programmable adenosine to inosine RNA editing, **(B)** dCas13 tethered to ADAR2dds for a single-nucleotide base editing in RNA. The dCas13 effector is fused with deaminase acting on RNA2 (ADAR2dd) for programmable Cytosine to Uracil RNA editing, **(C)** dCas13 tethered to a polyadenylation site or a polyadenylation factor. This system uses a dCas13 effector fused with an alternative splicing factor or AAUAAA polyadenylation sites to block access by the polyadenylation machinery. Depending on the positions of the polyadenylation site, it could be used to include or exclude specific exons during pre-mRNA processing, **(D)** dCas13 tethered to a splicing factor. This system could be used to characterize the functions of RNA isoforms of a specific gene, and **(E)** Cas13 system for rapid detection of *Mycoplasma* sp. as a contaminant in *Plasmodium* culture. CRISPR-Cas13 can be used to target *Mycoplasma* sp. AfterT7 transcription and amplification of target *Mycoplasma* sp. RNA, the transcript is subjected to CRISPR-Cas13 detection. The binding of crRNA to the target RNA activates the solvent-exposed HEPN site for non-specific cleavage of nearby RNA-linked reporters to emit a signal. The resulting signal could be measured using lateral flow detection with antigen-labeled reporters.

As earlier mentioned, >70% of *Plasmodium*’s genome remains functionally uncharacterized. It has indeed been posited that the sparse application of functionally characterizing genetic tools in *Plasmodium* forecasts that this proportion is unlikely to change if no new methods are developed (Le Roch et al., 2003). In light of this, the addition of the promising CRISPR-Cas13-based tools to the existing tool set for investigating the parasites’ genome will be immensely useful (Figure 5). Below we discuss the prospects of emerging Cas13 systems in alternative RNA editing and identify “hotspots” in *Plasmodium* research where they could be adapted.

#### 6.2.1. Hybrid Cas13-based systems as prospective tools for single-base editing in *Plasmodium*

Recent advances have seen the design of hybrid Cas13-based editing platforms capable of performing single-nucleotide editing in target locus. Generally, as shown in Figures 5A,B, these platforms make use of genetically engineered (mutated) Cas13 effectors (Cas13b or Cas13x.1) fused or tethered to other catalytic domains. One such application is RNA Editing for Programmable A-to-I Replacement version 1/2 (REPAIRv1 and REPAIRv2). The REPAIR systems introduce dead Cas13 effectors (Cas13 with a mutation in HEPN lacking RNase activity) fused with either endogenous exogenous adenosine deaminase acting on RNAs (ADARs) catalytic domain, which substitute adenosine for inosine into target transcripts and thereby stalls translation (Cox et al., 2017). Following the advent of the REPAIR systems, a new platform, RNA Editing for Specific C-to-U Exchange (RESCUE) system and its variants which performs both A-to-I and C-to-U RNA editing have been designed and applied to several genes (Abudayyeh et al., 2019; Li et al., 2021). These systems utilize the fusion of Cas13b with ADR2dd to create synergistic effectors with precise single-base editing capabilities (Abudayyeh et al., 2019; Li et al., 2021).

Although the development of these platforms is still at the embryonic stage (Li et al., 2021; Tang et al., 2021), they may be useful for the study of single-nucleotide polymorphism (SNP) variants and point mutations in *Plasmodium* if expanded and their efficiencies are improved. In *Plasmodium* parasites, there is a wealth of SNPs that could be used as proxies for genomic surveillance of drug resistance, and tracking the movement of *Plasmodium* strains on a global scale (Campino et al., 2011). For instance, a genome-wide integrated analysis of just three parasite strains from the Netherlands, Honduras, and Indochina unraveled 27,000 SNP of which many were uncharacterized (Miles et al., 2016). While few SNPs have been used to genotypically distinguish between parasites originating from different geographical locations, their phenotypic relevance is unknown (Campino et al., 2011; Bankole et al., 2018), suggesting the need to apply novel tools like REPAIR and RESCUE for characterization.

#### 6.2.2. Cas13, a prospective tool for characterizing alternative polyadenylation in *Plasmodium*

Alternative polyadenylation (APA) is conserved post-transcriptional machinery that promotes transcriptome and proteome diversity through the cleaving of alternative polyadenylation sites, followed by the adding of poly (A) tail to generate isoforms differing at the 3′ ends (Arora et al., 2022). APA shields mRNA from enzymatic degradation and regulates nuclear exports and translation (Zhang Y. et al., 2021). Depending on the site of cleavage, APA events could result in the inclusion or exclusion of exon (exon skipping) and intron retention (Yang et al., 2021) which consequently contributes to a diverse protein repertoire. Employing long-read sequencing techniques, Yang et al. (2021) identified 1,555 alternative polyadenylation sites in transcripts of asexual blood stages of *Plasmodium* parasites. Further results from this team indicated that 369 (23.7%) of the total transcripts harbored more than five (5) poly (A) sites. A different study showed the presence of AT-rich hexamer, AAUAUU, which putatively serves as a positive signal for polyadenylation in *Plasmodium falciparum* (Stevens et al., 2018). Nonetheless, the relevance of these APA sites and their corresponding isoforms remains largely unexplored in *Plasmodium* perhaps due to the unavailability of suitable tools for APA perturbation. Further, the types of APA (tandem 3’ UTR APA, alternative terminal exon APA, intronic APA, internal exon APA) events in *Plasmodium* remains largely unclassified. Recently, as shown in Figure 5C, Tian et al. (2022) developed a CRISPR-dCas13 system (CRISPR iPAS) that can be used to functionally characterize APA sites. They achieved this by combining EGFP-tagged dCasf13b from *Porphyromonas gulae* with crRNA-targeting upstream UGUA element or AAUAAA polyadenylation sites core regulation region to block access by the polyadenylation machinery. They demonstrated that, in HEK293T cells, CRISPR iPAS could regulate tandem 3’UTR, alternative terminal exon, and intronic polyadenylation site APA types with high specificity and efficiency. This system could be harnessed to study isoforms of genes that modulate drug resistance, transcriptional and post-transcriptional modification, and cellular differentiation in *Plasmodium* parasites.

#### 6.2.3. Cas13, a prospective tool for characterizing alternative splicing in *Plasmodium*

As established, alternative splicing plays an essential role in stage-specific cellular differentiation and the transition of the *Plasmodium* parasite from the sexual stage to the asexual stage and vice versa (Yeoh et al., 2019; Yang et al., 2021). It has been demonstrated to be a common phenomenon that usually controls sexual dimorphisms in *Plasmodium* species. Alternative splicing results in the generation of many different matured mRNA transcripts from a single pre-mRNA transcript; creating an important layer of regulation at the RNA level (Iriko et al., 2009). Indeed, a myriad of investigators had sought to quantify and explore the role of alternative splicing in essential genes of *Plasmodium* using RNA sequencing techniques (Sorber et al., 2011; Gabriel et al., 2015; Turnbull et al., 2017; Yeoh et al., 2019). Excitingly, recent advances in Cas13-based systems have seen the development of programmable mRNA splicing tools that can be used to efficiently characterize splicing events in *Plasmodium.* Generally, these Cas13-based splicing systems are designed to fuse dCas13 effectors with distinct splice elements for isoform perturbation (Figure 5D). *Via* this approach, *Konermann* and co-workers achieved >80% exon exclusion efficiency in human cells using multiple crRNAs targeting intronic branch point, splice acceptor site, exonic splice enhancer, and splice donor (Konermann et al., 2018). Interestingly, this system was adopted differently by Du et al. (2020). In their study, they successfully engineered a CRISPR artificial splicing factor by fusing dCas13 orthologs with splicing regulatory domains. They designed two crRNA that simultaneously target different positions on the sequence element which efficiently excludes or includes exons of interest during the processing of pre-mRNA. These programmable systems could be adapted to interrogate the role of alternative splicing in ApiAP2 transcriptional factors and how they contribute to the stage-dependent functions of ApiAP2 members in *Plasmodium* species (Quansah et al., 2022). Conceptually, these systems could be adapted for a bulk interrogation of alternative splicing targeting many isoforms of ApiAP2 genes in a single assay.

#### 6.2.4. Cas13, a prospective tool for detecting contaminants in cell cultures

Contaminants such as *Mycoplasma* usually remain inconspicuous in continuous *Plasmodium* cultures but substantially alter the property and behavior of infected cells making them potential significant confounders in experimental studies (Malave-Ramos et al., 2022). There is the prospect of repurposing the off-target property of Cas13 for detecting contaminants in long-term *in vitro Plasmodium* cell cultures (Figure 5E). Cas12a system has already been applied in *Mycoplasma* as a diagnostic tool proving the applicability of the CRISPR-Cas system in *Mycoplasma.* The relatively robust collateral activity of Cas13 over Cas12 makes it a more robust tool for the detection of *Mycoplasma* contaminants in *in vitro Plasmodium* cultures.

## 7. Conclusion

This review has summarized the discovery, classification, principle of action, and diagnostic platforms of CRISPR-Cas13 system. Further, it has shown that Cas13-based systems could be prospectively used for single-base editing, and characterizing alternative polyadenylation or alternative splicing in *Plasmodium* and detecting contaminants in *in vitro Plasmodium* cultures. The promising CRISPR-Cas13-based tools will be immensely useful for investigating the parasites’ genome.

## Author contributions

CZ: conceptualization. YC, EQ, CZ, and LY: writing–original draft. CZ, LY, DS, YC, EQ, SY, JW, YZ, and MC: writing– review and editing. All authors contributed to the article and approved the submitted version.

## Funding

This work was supported by the National Natural Science Foundation of China (82072304 and 81871671 to LY), grants from Scientific Research of BSKY from Anhui Medical University (XJ201807 to CZ), and the Foundation of Education Department of Anhui province (KJ2021A0213 to CZ).

## Conflict of interest

The authors declare that the research was conducted in the absence of any commercial or financial relationships that could be construed as a potential conflict of interest.

## Publisher’s note

All claims expressed in this article are solely those of the authors and do not necessarily represent those of their affiliated organizations, or those of the publisher, the editors and the reviewers. Any product that may be evaluated in this article, or claim that may be made by its manufacturer, is not guaranteed or endorsed by the publisher.
